# Design of a nursing psychoeducation program to reduce preoperative anxiety in adults

**DOI:** 10.3389/fpubh.2024.1391764

**Published:** 2024-06-03

**Authors:** Palmira Oliveira, Regina Pires, Rosa Silva, Carlos Sequeira

**Affiliations:** ^1^Nursing School of Porto (ESEP), Porto, Portugal; ^2^Center for Research in Health Technologies and Services (CINTESIS@RISE), Porto, Portugal

**Keywords:** adult, anxiety, nursing, preoperative period, program development, psychoeducation

## Abstract

**Introduction:**

Preoperative anxiety, with its multifactorial origins, affects a wide range of surgical patients, leading to adverse physiological and psychological effects in the perioperative period. Customized, autonomous nursing interventions are needed to address individual person needs. The shift toward outpatient surgery emphasizes the need for restructured nursing approaches. Existing literature suggests that preoperative nursing consultations offer opportunities for assessing needs, providing information, and prescribing anxiety-reduction strategies. Psychoeducation, a specialized skill within mental health and psychiatric nursing, has proven effective in alleviating preoperative anxiety and reducing postoperative complications. The aim is to obtain and analyze the information reflecting nurses’ understanding of the design, structure, and operationalization of a psychoeducation program to reduce preoperative anxiety in adults.

**Methods:**

A qualitative, exploratory, descriptive study was conducted. Data were collected through a 90-min focus group session held online via Zoom Meetings videoconferencing platform. Inclusion criteria for the participant’s selection were established. The focus group was guided to deliberate on potential strategies for crafting effective psychoeducational interventions. Data collection ceased upon reaching theoretical saturation and gathered information was submitted for content analysis. Ethical procedures were ensured.

**Results:**

Of the participants, 10 were specialist nurses (7 working in mental health and psychiatric nursing and the remaining in medical-surgical nursing), with an average age of 41 and an average of 15 years working in surgical services. The nurses selected the target population, the structure and content of the psychoeducation sessions, and the resources and addressed the perceived importance, effectiveness, and feasibility of the designed psychoeducation program.

**Discussion:**

The study revealed the nurses’ understanding of the design of a psychoeducation program potentially effective in reducing preoperative anxiety in adults, in an outpatient surgery context. This result will allow the transfer of the produced knowledge to nurses’ professional practice reflecting lower levels of anxiety and promoting a better surgical recovery. This is an unprecedented study conducted in Portugal, adding substantial knowledge to the nursing discipline. However, further research into implementing psychoeducation in a surgical context is suggested aiming to consolidate the results of research already carried out internationally.

## 1 Introduction

Currently, there is a paradigm shift in surgical care with the development of outpatient surgery since it allows for a reduction in waiting times for surgery and hospital stays. This provides less exposure to infections, and a rapid return to daily and social activity, thus contributing to the individualization of care (1) and a reduction in hospital costs (2). In contrast, this also implies less contact with health professionals, which can contribute to increased anxiety for the person undergoing surgery and hinder the continuity of care provided by nurses (2).

The literature shows that surgical intervention is perceived as an external threat and a trigger for anxiety (3). It is estimated that more than 50 per cent of people experience some level of preoperative anxiety (4, 5), and in Western countries, the prevalence of preoperative anxiety is estimated at 70 per cent (6).

Preoperative anxiety generates adverse physiological and psychological effects (7, 8), negatively impacting the perioperative period, for example, nausea, vomiting, fatigue, increased blood pressure, tachycardia, pain, high levels of inflammation, respiratory problems, delayed healing of surgical wounds, and increased morbidity and mortality rates (4, 5, 7, 9–13). Similarly, evidence shows that the highest level of anxiety occurs on the day of surgery (14) and that controlling anxiety reduces the dose of anesthesia and analgesia required (15, 16).

Anxiety is an emotion, a warning sign, an anticipation of a future threat (17). Therefore, it is considered a person’s natural reaction to potential threats, motivating the search for coping strategies. However, the intensity and meaning attributed to anxiety differ for each person, and if it exceeds the person’s reaction capacity, it can lead to serious complications. It is a unique and multifactorial experience and, thus, a challenge when prescribing individualized nursing interventions.

Spielberger (18) defined a theory that distinguishes between anxiety as a personality trait and anxiety as a transitory emotional state. Trait anxiety refers to a relatively stable state, corresponding to the individual’s baseline level of anxiety. State anxiety is a complex emotional reaction evoked by a stimulus perceived as a danger or threat to the individual.

The same author (18) also developed the STAI (State–Trait Anxiety Inventory), which is referred to as the gold standard for measuring anxiety levels in the surgical context (3, 7, 12, 13), validated for the Portuguese population by McIntyre and McIntyre (19). Notably, using anxiety assessment instruments on admission to the surgical service allows identifying people with high anxiety levels and developing preventative mitigation interventions (20). On the other hand, knowing the factors associated with higher anxiety levels helps define patients who can benefit from these preventive interventions.

There is no consensus on these risk factors, although variables such as gender, age, education, comorbidities, previous surgical experience, previous anesthesia, use of anxiolytics, waiting time, family dysfunction, level of knowledge about the procedure, type of surgery, psychiatric pathology, and anxiety disorders emerge as potential predictors of anxiety levels (7, 21).

According to the above-mentioned, coupled with the fact that high levels of anxiety can lead to the cancelation of surgeries (22), nurses play a key role in implementing autonomous and personalized interventions aimed at early diagnosis and intervention to reduce preoperative anxiety (12, 23), since the most common interventions include anxiolytics and sedative medication (24). Hence, nurses must reprogram care delivery to meet the real needs of ambulatory surgical patients (25) undergoing a health-disease transition process (16). This process differs from person to person and requires qualified and individualized care to reduce anxiety levels, with particular emphasis on preoperative nursing care, namely the nursing visit/consultation (26).

Several studies show that preoperative nurse consultation is a crucial time to provide information and diagnose needs by establishing a specific nursing care plan that helps reduce anxiety (2, 7, 21, 27, 28) and, consequently, reduce possible complications (26).

In addition, nurses play a crucial role in implementing interventions to reduce preoperative anxiety, as they are trained professionals in direct contact with the patient from admission to discharge (12), allowing them to use various strategies such as effective communication with patients, education about procedures, pain management and postoperative care, relaxation techniques, creating a calm and relaxing environment, and providing emotional support throughout the process.

On the other hand, nurses are encouraged to develop models for providing information in the preoperative period to reduce anxiety (29). This is essential because, in Portugal, many patients have little or no information about surgical procedures, and most health institutions do not have consistent preoperative preparation protocols (8).

Considering the above stated, there is a consensus that preoperative education on anxiety management strategies can reduce preoperative and postoperative anxiety levels and consequent complications and that intervention strategies include psychoeducation, information associated with surgery, and relaxation techniques (30, 31).

The term psychoeducation was first used in 1980 for treating patients with schizophrenia (32). Since then, psychoeducation has been used as a systematic and structured intervention in different health contexts, such as cardiovascular disease, oncological disease, and dementia, among others (33).

Psychoeducation can be defined as educating a person about the symptoms, treatment, and prognosis inherent in a health condition. However, more than providing information, it is essential to promote the person’s awareness and involvement, empowering them with skills and providing tools to help them manage, cope with, and live with their condition, thus facilitating changes related to attitudes and behaviors (34, 35) to attain optimal health and well-being.

Psychoeducational intervention can have different focuses, for example, compliance/adherence, illness, treatment, and rehabilitation (36), allowing for prevention, promotion, and health education (37). It involves emotional support, including empowering people, managing expectations and emotions (38), helping change the meanings of mental disorders, and integrating psychotherapeutic, didactic, and systematic interventions to inform users about treatment and pathology.

Psychoeducation combines elements of cognitive-behavioral therapy, group therapy and pedagogy (36). Recent studies show that psychoeducation has been mostly applied with other psychosocial therapies rather than as an isolated therapy (39). It can be developed in a group or individually (37), with the individual modality favoring a personalized approach according to each person’s needs (40). However, this approach is more time-and cost-consuming, although it allows people to express themselves more easily (39). Group psychoeducation facilitates dynamic interaction between group members by sharing problems, feelings, experiences, ideas, and reactions (39).

Psychoeducation models vary according to the target group and the psychoeducation focus. An information model, a skills training model, a support model, or a more comprehensive approach can be used (36). The information model focuses on knowledge about the illness and its management, while the skills training model centers on developing self-competencies for more effective management of illness. The support model is focused mainly on the help of support groups to encourage the patient’s family members to share their feelings. The most comprehensive model combines the various models to respond to the needs of users and their families/carers (33, 36).

Also, psychoeducation can be active or passive. In active psychoeducation, the psychoeducation facilitator is actively involved with the person/family, leading to interaction. In passive psychoeducation, the individuals/family members are provided with pamphlets and audio/video material to read and assimilate information independently (36).

The facilitator can have either a paternalistic or a collaborative approach. The paternalistic approach considers the knowledge transmitted by the facilitator without considering the person’s preferences, while in the collaborative approach, there is a dialog between the professional and the person in a co-construction approach aimed at reflecting on the particularities of their experiences (33).

Therefore, the design of psychoeducational interventions can vary according to the context, the facilitator, the number of sessions, and group or individual, among others. Deciding on the best methodology depends on the individual’s needs, the content, the existing resources (time, materials, and human resources), and the objective to be achieved (33). The structure of the psychoeducational program is usually organized as systematized, pre-planned multi-sessions, following a deductive alignment starting with theoretical content and ending with training in daily life skills (33). Psychoeducation usually comprises 5 to 24 sessions, lasting 40 to 60 min each, mainly weekly (36). Nevertheless, the literature is not consistent since some studies suggest different designs, with brief psychoeducation emerging as a new concept. According to Zhao et al. (41), brief psychoeducation is a short period of psychoeducation, including 10 sessions or less. Brief psychoeducation can improve overall condition in the long term, promote improvement in mental state in the short term, and reduce the incidence and severity of anxiety in the medium term; however, the usefulness of brief psychoeducation remains questionable (41). On the other hand, the average duration of psychoeducation is around 12 weeks(41).

Regarding anxiety management, psychoeducation is a relevant intervention, supported by its positive effects on anxiety relief (30, 33, 42).

Gomes and Pergher (43) also believe that psychoeducation should be used as a form of surgical psychoprophylaxis in pre-and post-surgical follow-up for patients undergoing cardiovascular surgery. The authors concluded that obtaining information about the disease and treatment reduces anxiety and that in addition to information, relaxation is another resource commonly used with hospitalized patients because it reduces anxiety, stimulates self-care, and improves motivation for treatment (43). The same authors stress that Jacobson’s progressive muscle relaxation and breathing training can be combined with guided imagination, autosuggestion, and distraction exercises, encouraging their use in the surgical context (43). Other studies, including psychoeducational intervention and relaxation practice, show a reduction in stress and an improvement in psychological health, including anxiety symptoms (44).

Considering the lack of national and international publications on psychoeducation programs to reduce preoperative anxiety in adult outpatients (3), it is pertinent to design a psychoeducation program in nursing with this objective and evaluate its impact. The specialist nurse in mental health and psychiatric nursing is responsible for the psychoeducational intervention in Portugal (45).

Thus, the present study is part of a broader research project, which includes three tasks: 1-A study of the scientific evidence on psychoeducation programs on preoperative anxiety in adults, which has already been carried out (3); 2-Design of a nursing psychoeducation program to reduce preoperative anxiety in adults (currently under development); 3-Evaluation of the effectiveness of the nursing psychoeducation program in reducing preoperative anxiety in adults, which will be conducted by a randomized controlled clinical trial.

This present study refers to task 2 of the broader research project. Thus, the objective of this task is to design a nursing psychoeducation program aimed at reducing preoperative anxiety in adults.

## 2 Materials and methods

This study was a qualitative, exploratory-descriptive study. Data was collected through a focus group (FG) session to understand nurses’ opinions on the design, structure, and applicability of a psychoeducation program. The qualitative method, more specifically focus groups, was considered a methodologically appropriate strategy to delve deeper into the subjective experiences and perceptions of nurses. This method enables research based on the naturalistic paradigm, which values the meaning or nature of the experience lived by participants in a specific context, the understanding and interpretation of the phenomenon under study (46). Therefore, allows for highlighting the experience and skills of each nurse within a surgical context. FG facilitate dynamic discussions that can reveal diverse opinions and subtle nuances, crucial for effectively developing and adapting the psychoeducation program to meet the specific needs of people facing preoperative anxiety. The planning of the session was supported by the generic objectives of the extended project.

Considering that the size of FG can vary between four and 12 participants (46), 12 nurses were recruited (via email and/or telephone) to participate in the FG, selected from the contact network of the researchers of this study (47).

To recruit an accessible, diverse sample with advanced skills and professional experience in the field of surgery and/or mental health, the inclusion criteria were (i) nurses specialized in mental health and psychiatric nursing or medical-surgical nursing, (ii) having experience in the field of surgical patient care (inpatient or operative room).

Prior approval was obtained from the ethics committee (ethical approval number: 03/2023) to conduct this study.

### 2.1 Operationalization of the focus group

The FG was held online via the Zoom Meetings videoconferencing platform on 28 April 2023 and lasted 90 min.

The FG session began with a presentation of the broader research project. Then, the context in which the psychoeducation program will be implemented was presented (the Ambulatory Surgery Unit - UCA) of an institution in the north of the country, which has a preoperative nursing consultation for patients scheduled to undergo certain types of surgery).

This was followed by a theoretical presentation on the concept of psychoeducation and the results of the scoping review carried out in the project’s first study, which aimed to map knowledge about existing psychoeducation programs to reduce preoperative anxiety in adults (3).

Nurses were asked to complete a socio-professional characterization questionnaire, and after being informed of the rules of the FG and its objectives, they signed an informed consent form.

The discussion of ideas during the FG was organized based on a list of topics previously formulated and validated by the members of the project’s research team (see Table 1). During the FG session, all participants had the opportunity to communicate freely, spontaneously, and with motivation, and there was a comfortable atmosphere among the participants during the FG.

**Table 1:** Topic list of the focus group.

Interview topics	Questions
Target population	1-In addition to the following inclusion criteria: adult individuals and presenting preoperative anxiety symptoms, what other criteria should be defined for the target population? 2-Should the person undergoing surgery be accompanied during the sessions? If so, who should it be and what inclusion criteria should be defined?
Structure of the psychoeducation sessions	1-Considering that the psychoeducation program will be implemented in the preoperative consultation of an UCA (Ambulatory Surgery Unit) how many sessions should the program include? 2-Should the sessions be held face-to-face, online, both or other way? 3-Are the sessions to be held individually or with a group of people? If with a group, how many people should the group have? 4-In which moment of the perioperative period should the sessions be implemented? 5-What is the duration and frequency of each session? 6-How should the sessions be monitored?
Content of the psychoeducation sessions	1-What content should be discussed during the sessions? In what sequence? 2-What psychometric instrument should be used to assess anxiety? When should it be applied? 3-What nursing interventions should be implemented to reduce anxiety? 4-What activities can operationalize the nursing interventions? And what method should be used (expositive, demonstration, other)?
Resources	1-Who should organize the sessions? And how many facilitators should be there? 2-What material resources (audiovisuals, leaflets, others) can be used during the sessions? When should they be used? 3-Besides the instruments to assess anxiety levels, what other psychometric instruments should be used and when?
Perception of the importance, efficacy, and feasibility of the psychoeducation program	1-What is your opinion of the importance, effectiveness, and feasibility of the newly designed program?

The session was videorecorded, and the information was transcribed verbatim and then submitted to content analysis (48). The data were coded independently by two researchers (PO and RP). The results were interpreted and compared, and differences were discussed until a consensus was reached.

At the end of the session, all participants considered that there was no new information about the psychoeducation program that required analysis/discussion, and theoretical saturation was considered to exist, as the mapping of the themes analyzed adequately explained the psychoeducation program. Additionally, information saturation was also validated by peer analysis, that is, by a second researcher who participated in the FG through its content analysis. On the other hand, research was carried out exhaustive research on the phenomenon under study, checking the density of the data obtained and finding no new explanations, interpretations, or descriptions of the phenomenon, in addition to the fact that the FG was supported on the results of the scoping review and, included experienced participants in the surgical context and in mental health, therefore, it was considered that the criterion of saturation was reached.

In operationalizing of the FG, it should be noted that the methods and strategies inherent to qualitative methodology were adopted in the information collection and processing processes, so other elements of the trustworthiness were also considered. Concerning the credibility, we sought to faithfully translate reality, resulting from the ability to establish an empathetic relationship with participants, maintaining an active listening stance and reflection on situations, as well as emotional distancing from the information provided. Reliability validation was achieved by directly questioning participants about the meaning of what they mentioned and by final analysis of the participants’ program design, and still, we detailed the methods and procedures, enabling the study to be reproducible within similar contexts. Regarding transferability, our objective was not to produce generalizations but to understand and gain knowledge about the problem under study in a specific context. However, we believe these study findings find similarities in identical ecologies and circumstances and can contribute to analytical generalizability, and there is potential for it to be transferable.

## 3 Results

Of the 12 invited nurses, 10 were able to participate in the FG. The most defiant topics were related to the number and frequency of sessions and how to operationalize the content to be discussed in each session.

### 3.1 Characterization of the participants

Nurses were aged between 26 and 54, with an average age of 41, and only one was male. On average, they had been working for 19 years, and in surgery settings for 15 years. All nurses have experience in the surgical context and in providing care to people with pre-operative anxiety. They are also specialist nurses, with seven nurses were specialized in mental health and psychiatric nursing, and the remaining in medical-surgical nursing. This means that most nurses have advanced knowledge and skills in anxiety reduction strategies, experience in clinical practice in a surgical context, and have carried out preoperative anxiety management interventions.

### 3.2 Psychoeducation program to reduce preoperative anxiety in adults

The results are presented in five sections, according to the topics that guided the discussion for the construction of the psychoeducation program, using the participants’ verbatim (Table 2).

**Table 2:** Design of the psychoeducation program to reduce preoperative anxiety in adults: illustrative quotations.

Topic	Subtopic	Illustrative quotations
Target population	Inclusion and exclusion criteria	P2- “for the patients to be included, besides being adults and presenting anxiety, they cannot have been diagnosed with any mental disorder; or rather, they must not have changes in cognition.” P5- “no cognitive or behavioral changes, like agitation.” P6- “knowing how to read and write.” P8- “not feeling confused.” P9- “the patient’s interest in participating and getting involved in nursing interventions aimed at reducing their anxiety should be assessed.” P10- “if the patients meet the conditions we are listing, there is no need for them to be accompanied, because then we would have difficulty in defining criteria for them and they might not even be available to attend the sessions.” P1- “The sessions should be individual, because we have to personalize the strategies according to the level of anxiety, type of surgery, previous surgical experiences, level of literacy, etc., and in my experience, individual sessions are more effective than group sessions.” P3- “although group sessions demand less resources, individual sessions allow empathy to be established and favor the therapeutic relationship.” “I would say: No to the companion.”
2-Structure of the psychoeducation sessions	Number, periodicity, operationalization, duration	P5- “at least 3 sessions” P4- “I also agree that the ideal would be at least 3 sessions, but we can adjust this with the number of times the patient comes to the hospital before the surgery.” P1- “right then, when they come for an anesthetic consultation, then they should have the preoperative nursing consultation and the first session should be done in person.” P7- “the third session should be on the day of the surgery or the day before if the patient comes to the hospital, in person.” P3- “the middle session can be done over the phone or via online computer platforms, if the patient is able to do so.” P2- “I agree, via telephone or online, and the date will depend on how long it takes between the anesthetic appointment and the surgery day, and it should take place between those times, so it could be once a week or even twice, or there could be a longer interval between sessions.” P1- “the preoperative nursing consultation in the UCA (Ambulatory Surgery Unit), lasts currently about 45 min.” P2- “in my institution there is no preoperative nursing consultation.” P9- “as far as we know, psychoeducation sessions last about 45 min, so considering what is currently done in the preoperative consultation, the first session should be at least 60 min to a maximum of 90 min, and sessions 2 and 3 can be shorter, depending on the patient’s needs.”
3-Content of the psychoeducation sessions	Topics per session, tables/sequence of topics, typology of strategies for reducing anxiety	P8- “I think that in the first session we should assess whether the patient meets the inclusion criteria defined, and assess the level of anxiety, and since the results presented here from the scoping review used the STAI, and since it is translated for the Portuguese population, I think it would be a good option!” P5- “in addition to anxiety, in the first session we should also assess the level of anxiety self-control, and I know that there is a validated NOC (nursing outcomes classification) that we can use.” P6- “all the information related to the surgery, pre-and post-operative nursing care and support networks should be transmitted, and ideally this should be done by the specialist nurse in medical-surgical nursing.” P2 - “I think pre-and post-operative teaching is essential, because only after the patient is sure of what is going to happen in surgery will they be able to hear and retain something related to strategies for reducing anxiety.” P4 - “it’s important to talk about the concept of anxiety, how it can be recognized and its implications for health, both in the preoperative period and in the postoperative period.” P10- “as for strategies to reduce/manage anxiety, I think this should have the intervention of a specialist in mental health and psychiatric nursing.” P1- “So the ideal thing would be for there to be a specialist nurse in mental health and psychiatric nursing in the outpatient department, who would be available on the days of the preoperative nursing consultation, to intervene if necessary, and could support the consultation.” P3- “in this first session, you can teach, instruct and, if possible, train the patient in strategies for self-control of anxiety, and play a video (from YouTube, or made by the ward nursing team).” P2- “yes, I think so! and that the patient takes this video home, to continue training at home and in the second session we would telephone or make a video call, or by a computer platform, to find out if the patient has been using these strategies, and what doubts or difficulties they have had.”
		P4- “and if the patient is not able to use the video or computer, and this also depends on the age group, we can use a leaflet.” P1- “a digital spot with a hyperlink could also be a possibility.” P5- “more important than a leaflet, seeing something real is important in a program like this.” P7- “I would add that in the second session via telephone or online, in addition to assessing aspects related to strategy training, you can also assess the patient’s perception of the evolution of their level of anxiety, as well as signs and symptoms, without using a psychometric instrument.” P9- “yes, we can even teach the patient to record their progress at home, in other words, to keep an anxiety diary of their perception.” P5- “I suggest that in the third session, the patient practices the anxiety reduction/management strategies again and that we assess the patient’s level of anxiety and self-control again with the STAI.” P10- “as for the strategies that the patient can use at home, although modified muscle relaxation is effective, it seems a little complex for the patient to learn to practice at home on their own.” P3 - “the patient can learn to do muscle relaxation and although most of us think that the patient would not do it at home, I think it’s important to address it because of its effectiveness.” P5 - “I suggest square breathing and adapted mental imagery.” P2 - “diaphragmatic breathing, even at bedtime, the patient can do that.” P1- “guided imagination works very well for some people, but not others, because they cannot do it and would have to practice more.” P8- “the patient should have the opportunity to choose which strategy to use, and can even combine distraction, such as reading, physical exercise, music, and aromatherapy.” P7- “yes, the patient will have the videos or the leaflets that we will provide at the first session.”
Resources	Individual, materials, psychometric	P3- “from what we have said, the program should be run by a specialist nurse, ideally in medical-surgical nursing and the other in mental health and psychiatric nursing.” P4- “we know it will be difficult to have two specialist nurses, but we should have at least one, although the psychoeducation program should be implemented by a specialist in mental health and psychiatric nursing, according to our legislation.” P10 - “Since the nurse specializing in mental health nursing who will facilitate the program must reveal expertise in the area by having at least two consecutive years of clinical experience in surgery.” P10- “as material resources, we talked about the video and/or pamphlet with strategies for reducing anxiety.” P9- “as for the psychometric instruments to be used, in addition to the STAI, we talked about assessing the level of anxiety self-control with the NOC and applying them in the first and third sessions.”
Perception of the importance, efficacy, and feasibility of the psychoeducation program		P5- “I think this program is clearly necessary… extremely important,… I think it’s an area of nursing intervention, I would say noble,… so it should already exist, but in terms of feasibility, resources for a program like we have designed here, I see difficulties, both in getting physical spaces, and in making nurses available to implement it… and in reconciling it with the user’s traveling to the hospital.., but yes!… we should start and use research!… and the results and health gains that can be obtained, which I think this program would achieve.” P8- “After listening to my colleagues, we concluded that this program proposal is complete, it is extremely important, and that it is possible to be implemented, benefiting the recipient of our care.” P10- “I think it’s an asset, but I really agree with the difficulties, but it’s not impossible!.”

### 3.3 Target population

The psychoeducation program is aimed at adults under 65, in a preoperative situation, who show anxiety, the ability to read and write, motivation to participate and signed an informed consent form. They must not have cognitive or behavioral disorders. The sessions are only aimed at the individual who will be submitted to surgery and meets the abovementioned criteria.

### 3.4 Structure of the psychoeducation sessions

The psychoeducation program should include three individual sessions. The first and third sessions will be in-person, and the second will be via telephone or computer platforms.

Session 1 will be held during the preoperative nursing consultation (60 to 90 min), and Session 2 will take place at an unspecified time (depending on the availability of the nurse and the patient and the timing between the anesthetic consultation and the surgery) and Session 3 on the day of the surgery. Sessions 2 and 3 can be shorter, and the duration will depend on the person’s identified needs.

### 3.5 Contents of the psychoeducation sessions

In Session 1, the specialist nurse in mental health and psychiatric nursing should sequentially assess whether the person meets the inclusion criteria for the program, the level of anxiety using the STAI in the Portuguese version (19), the level of anxiety self-control using the NOC (Nursing Outcomes Classification) “level of anxiety self-control” (49), teach about preoperative and postoperative care, clarify doubts, teach about anxiety, teach, instruct, and train anxiety management strategies (provide a video, pamphlet or link, to promote training at home). On the other hand, nurses consider that anxiety management strategies should be personalized (Table 2).

In Session 2, the nurse contacts the person by telephone or via a computer platform to assess the perception of the evolution of anxiety and its daily record, monitor the frequency of training and type of strategies used to manage anxiety and clarify doubts.

In Session 3, the nurse supervises the person adopting anxiety management strategies, assesses the level of anxiety using the Portuguese version of the STAI (19), and assesses the level of self-control of anxiety using the NOC.

Regarding the anxiety management strategies, the nurses suggested modified muscle relaxation, diaphragmatic breathing, square breathing, guided imagination, mental imagery, aromatherapy, and the use of distraction techniques (music, physical exercise, reading), so the person should be involved in selecting the strategies to be used, either isolated or combined.

### 3.6 Resources

A nurse specialized in mental health and psychiatric nursing will be conducting the psychoeducation sessions. This professional nurse who will participate in the three sessions has the appropriate training and skills while working at UCA. Moreover, this professional has clinical practice experience of at least 2 years in a surgical context (Table 2).

Video or pamphlets can be used as material resources.

In sessions 1 and 3, the STAI (19) will assess the level of anxiety and the NOC’s “self-control level of anxiety” (49).

### 3.7 Perception of the importance, effectiveness, and feasibility of the psychoeducation program

The nurses recognize the importance of designing a psychoeducation program and that the outlined program is potentially effective in reducing preoperative anxiety. However, they acknowledge the difficulty in implementing this program in current healthcare institutions. This difficulty is explained by the fact that this implementation requires conditions adapted to the program, such as human resources, materials, and the professional’s availability of time, coupled with the difficulty of the patient traveling to the hospital.

## 4 Discussion

In the context of outpatient surgery, the psychoeducation program to reduce preoperative anxiety in adults was designed according to the concept of psychoeducation and the structuring of an intervention program. This design was supported by the results of international research, and its development was conducted and supported in a scoping review (3) carried out for this purpose. Four studies were identified on psychoeducation programs to reduce preoperative anxiety in a hospital setting, with different characteristics, three of which showed a reduction in anxiety levels (50–52).

Notably, in the present study, the nurses prioritized the personalization of their interventions for the target population, stating that the sessions should be individual and mostly face-to-face, favoring the establishment of effective communication that supports the development of a therapeutic and trusting relationship between the nurse and the recipient of care. This is corroborated by other studies (13, 53), which state that people experience surgery differently, so individualized care based on the needs and personal characteristics of the patients is essential and should be considered when selecting the methods to manage anxiety and the quantity and quality of information and ways of providing this information (30).

Research shows that providing information can reduce the patient’s anxiety, and nurses play a crucial role in advising and informing; thus, these professionals need appropriate knowledge and skills (54, 55). In addition, preoperative education should be adapted to each person’s information-seeking styles since the evidence shows that individualized adaptation effectively reduces preoperative anxiety (13).

Thus, a face-to-face preoperative nursing consultation led to the creation of communication channels that stimulate expressing emotions such as anxiety, making it easier for the nurse to identify the person’s needs and enabling care tailored to them (21, 56). Nurses, and more specifically, the specialist nurse in mental health and psychiatric nursing, must develop communication techniques that facilitate interaction with the care client, and they can use interpersonal relationship theory to support their actions (57). This is favored by the organizational model associated with outpatient surgery, as it is person-centered, where face-to-face nursing consultations facilitate quality care (2), making it possible to draw up and implement an individualized nursing process. Nurses will be able to better understand people in the face of the transitions they experience, adopting the role of facilitator of a healthy transition (58), where the person must be empowered with information and have an active role in making decisions about their health (2).

Emphasis was given to the ability to read and write and the absence of changes in cognition, among others, when defining the inclusion criteria. These criteria have been cited in other studies stating that the information provided throughout the peri-operative course should be individualized, considering literacy and cognitive ability (59). On the other hand, other research shows that people with lower education levels are prone to being more anxious (60), likely related to difficulties in understanding the information provided and the lack of confidence in decision-making inherent to the process.

Several studies suggest that preoperative anxiety is present in all surgical patients, regardless of diagnosis (61). These studies also suggest that even in minor surgeries, patients experience high anxiety levels; therefore, all patients who undergo surgery should be targeted for intervention to avoid postoperative complications (62).

Regarding the structure of the psychoeducation sessions, the nurses opted for a short period of psychoeducation (three sessions), called brief psychoeducation (41). Research shows that this type of approach can reduce the incidence and severity of anxiety in the medium term, although the usefulness of brief psychoeducation raises some critical questions (41).

The sessions should be held individually and face-to-face, using the telephone or computerized platforms. The duration of the sessions varies according to the needs identified, with Session 1 being the longest, between 60 and 90 min. Similarly, the scoping review by Oliveira and colleagues (3) showed variations in the frequency, duration, and periodicity of sessions in international psychoeducation programs. Also, some studies applied a single-session intervention lasting on average less than 30 min (13).

Regarding the time for implementing the intervention, the nurses recommend that the first session be held during the preoperative nursing consultation associated with the anesthesia consultation and Session 3 on the day of surgery, with the intermediate session having no fixed time. There was also diversity in the studies included in the scoping review regarding the time interval between the moment of implementation and the date of surgery (3).

Concerning the context in which intervention programs are implemented, the need for a preoperative, face-to-face nursing consultation is highlighted (2, 21), enabling nurses to welcome the patient, assess their specific needs, and provide information related to surgery and preparation for discharge (25). Similarly, the studies included in the scoping review were conducted in an outpatient clinic or during hospitalization (3). In fact, Leal (63) proposes that the preoperative nursing consultation should take place 5 to 6 days before the intervention in the surgery department after the anesthesia consultation.

Regarding the content of the psychoeducation sessions, the nurses established the contents, sequence, and operationalization strategies, namely, what, and when to use the psychometric instruments. The Portuguese version of the STAI was used, similar to the study by Oliveira and colleagues (3). The suggested nursing interventions follow the scope of assessment, information, teaching, instructing, and training (64). Also, they include a pedagogical component about the aspects inherent to the surgical act, the concept of anxiety, and a psychic component oriented toward anxiety management strategies. The nurses also considered how the user’s training in anxiety management strategies should be monitored, stressing that they should participate in selecting each isolated or combined strategy, allowing for individualized and participatory care.

Concerning the anxiety management strategies, the nurses suggested interventions such as modified muscle relaxation, diaphragmatic breathing, guided imagination, and distraction techniques (music, physical exercise, reading), among others. These interventions, suggested by participants, are in line with what has been suggested by the best scientific evidence (3, 43, 65). In this context, systematic preoperative training by nurses, with the support of an information manual and relaxation strategies (muscle relaxation, deep and rhythmic inhalations), proved effective in reducing anxiety levels (66).

The study by Zhuo et al. (13) revealed music as the most used intervention to reduce preoperative anxiety, where patients could select the music; however, massage and video were also commonly used in adult patients. In fact, music is mentioned as a viable option instead of the use of sedative and anxiolytic medication, for reducing preoperative anxiety or at least reducing the need for these drugs (67). However, no nurse in this study suggested massage intervention as an option for inclusion in the program. This might be explained by the fact that most massages need to be performed by someone other than the patient, and the program was tailored only to the recipient of the intervention.

Concerning the resources needed for the psychoeducation program, the participants mentioned that it should be implemented by specialists (mental health and psychiatric nursing). They identified the psychometric instruments to be used and that the interventions could be supported with videos or pamphlets.

In Portugal, the competencies required of a nurse in the outpatient setting are the same as those required in the inpatient setting (25). However, patients have different needs; thus, somewhat different approaches are necessary (63). In addition, it is also crucial to carefully evaluate the identified needs, with the nurse playing a decisive role.

On the other hand, nursing consultations require nurses to have technical, human, and scientific competencies. These are defined in the competencies of the specialist nurse in medical-surgical nursing and comprise several complementing areas, particularly in the peri-operative consultation, where the specialist nurse is autonomous in managing the patient’s therapeutic process (68). The specialist nurse in medical-surgical nursing plays a fundamental role in empowering the person to properly manage the surgical experience, ensuring that they understand the information provided to favor the patient’s self-determination and decision-making (2).

It should also be noted that in the preoperative period, the nurse’s focus is preparing the patient physically and psychologically for surgery, providing them with the tools they need to manage their emotions (63). Therefore, alongside the role of the specialist nurse in medical-surgical nursing, the specialist nurse in mental health and psychiatric nursing emerges, who is the nurse who has the skills to implement a psychoeducation program. Psychoeducational interventions are commonly attributed to mental health nurses (45), aiming at empowering, and enabling people to manage and control their anxiety levels (69). The specialist nurse in mental health and psychiatric nursing is responsible for mobilizing the interaction dynamics with the patient; therefore, this professional is a crucial element in intervening and implementing psychoeducational interventions that promote knowledge, enable understanding and management of the disease process, and empower the patient to adopt appropriate strategies to cope with their living conditions, contributing to the maintenance, improvement, and recovery of their health (33).

Regarding material resources, many institutions use the information leaflet as a standard intervention, increasing the patients’ knowledge but not always reducing people’s anxiety levels because it does not respect their individuality, expectations, and fears (30). The present study adopted passive psychoeducation since the pamphlet is easy to implement and disseminate, enabling it to reach many people at a relatively low cost (70). Passive psychoeducation is common for patients experiencing anxiety, and in addition to the pamphlet, books or videos can be used to provide additional information about this condition (36). This allows the assimilation of the content transmitted verbally (37). However, the information should be as individualized as possible and meet the patient’s needs for quantity and quality (30).

As for the use of video, several studies describe it as an efficient and appropriate audiovisual strategy for transmitting information to patients, thus reducing anxiety levels (71).

Therefore, nurses advocate the use of passive psychoeducational interventions and active psychoeducational interventions (36) in their programs, using a collaborative approach (33).

The participants also expressed their perception of the importance, effectiveness, and feasibility of the psychoeducation program, stating that they are aware of the importance and relevance of its development and its impact on the delivery of nursing care and in reducing the patient’s anxiety levels. However, they also identify difficulties in its implementation, mainly related to material and human resources and nurses’ time availability.

In Portugal, surgery is primarily performed in an outpatient setting (2), so developing adapted professional practice and more research in this context is recommended. However, according to Leal (63), in the Portuguese context, the reduction in hospitalization stays means that nurses have less time to invest in pre-and postoperative care, increasing the financial burden derived from the incidence of late complications due to the patient’s lack of knowledge. The study by Lopes (2) on face-to-face nursing consultations for people undergoing outpatient surgery identifies some difficulties in its implementation, overlapping some of the difficulties expressed by the nurses in this study. For example, the patient’s travel to the consultation site, the lack of adjustment of parameterization and organization of the consultation, the training and time availability of the nurses, inadequate physical space, and a lack of human resources are mentioned.

Despite the difficulties expressed by the nurses, the feasibility and effectiveness of the psychoeducation program are perceived as positive, and the advantages are recognized since less anxious patients are more prone to cooperate, reducing surgery time, the need for drugs, and the number of surgical complications (27).

Non-pharmacological approaches to reducing anxiety, such as psychoeducation, are cost-effective, minimally invasive, and have a low risk of adverse effects (13). However, one of the major obstacles to implementing and using psychoeducation is likely the lack of knowledge and skills in its execution due to a lack of training opportunities (72, 73). In addition, nurses need specific training in strategies to reduce preoperative anxiety (66). Therefore, to implement anxiety management strategies effectively, and more specifically, regarding the necessary training, the specialist nurse in mental health and psychiatric nursing has the academic training and regulated skills (44). Concerning the resources, it should be noted that it will be necessary to provide an appropriate setting specifically identified for this purpose, having the necessary equipment (e.g., couch) to implement each strategy to reduce anxiety and ensure that the specialist nurse in mental health and psychiatric nursing has time available to implement each session. But the human resources are guaranteed, as the UCA has more than one nurse specialist in mental health and psychiatric nursing, with over 2 years of clinical practice in surgery. Regarding the necessary support, the hospital organization where the nursing psychoeducation program will be implemented, demonstrates interest through the institutional policies adopted and recognizes the clinical potential in health gains for the surgical client by investing in and valuing this type of intervention. So, we consider that the conditions for implementing the psychoeducation program are met, namely with authorization from the ethics committee and the UCA Clinical Director.

The knowledge that has emerged from this study supports the implementation of the last stage of the broader research project, aiming to evaluate the effectiveness of the psychoeducation program, providing a significant contribution to the knowledge of nursing as a discipline. It will support the development of the competencies required for nurses’ care practices in surgical settings, where specialist nurses in mental health and psychiatric nursing play a crucial role in reducing the emotion, anxiety. Most importantly, helping reduce/manage the anxiety levels of the recipient of care will enable the transferability of knowledge to nurses’ professional practice.

### 4.1 Limitations of the study

Despite nurses’ extensive professional experience in surgical settings and being familiarized with the topics under discussion, they originate from different hospital institutions with specific realities. This can enrich the discussion by sharing different perspectives. However, the fact that some of the nurses do not have a preoperative consultation established or the same functioning, especially concerning outpatient surgery (complexity of the surgeries, consultation scheduling, among others), may have also hindered the ability of some nurses putting into perspective the implementation of the outlined psychoeducation program.

### 4.2 Implications for practice and future research

Notably, the design of the newly constructed psychoeducation program was developed by nurses experienced in surgery and mental health and psychiatric nursing, aiming at its implementation by nurses in different outpatient surgery settings. But it could be complemented or extended to other professionals within a multidisciplinary health team.

Thus, nurses are actively involved in interventions to reduce preoperative anxiety in adults. However, despite the advantages of being a personalized design and an unprecedented advance in research in Portugal, comparing this study to other studies can be challenging. Scientific evidence has shown that psychoeducation as a health intervention produces positive results manifested in multiple ways, impacting the individual/family’s quality of life. However, comparing, and systematizing results poses some problems deriving from the diversity of methodologies used in research (40).

Therefore, research must focus on effective non-pharmacological strategies to reduce preoperative anxiety, particularly the effectiveness of psychoeducational interventions, which are less expensive than pharmacological interventions (70). In addition, nurses should invest in psychoeducation and anxiety management strategies training.

In light of the above-mentioned, a new study is planned to evaluate the effectiveness of the designed psychoeducation program, expanding the search for evidence on the effectiveness of psychoeducational programs, considering their specific content, the duration of the psychoeducational intervention, and the evaluation methodology.

On the other hand, these study results will support nurses’ clinical practice in a surgical context, boosting health gains for people undergoing surgery. Moreover, these outcomes may raise awareness of the need for specialist nurses in mental health and psychiatric nursing to practice their skills. This will enable the individual to experience the transition processes associated with surgery in a healthier way, promoting more adaptive responses and enhancing their quality of life.

## Data availability statement

The original contributions presented in the study are included in the article/supplementary material, further inquiries can be directed to the corresponding author.

## Ethics statement

The studies involving humans were approved by The Comissão de Ética da Sociedade Portuguesa de Enfermagem de Saúde Mental (ethical approval number: 03/2023) approved this study on 14/03/2023. The studies were conducted in accordance with the local legislation and institutional requirements. The participants provided their written informed consent to participate in this study.

## Author contributions

PO: Conceptualization, Data curation, Formal analysis, Funding acquisition, Methodology, Project administration, Resources, Visualization, Writing – original draft, Writing – review & editing. RP: Data curation, Formal analysis, Methodology, Project administration, Resources, Validation, Visualization, Writing – original draft, Writing – review & editing. RS: Project administration, Visualization, Writing – review & editing. CS: Conceptualization, Funding acquisition, Methodology, Project administration, Resources, Validation, Writing – review & editing.
